# Photovoltaic-Related “Black Swan” Hypothesis for Electric Power System: Phenomenology, Simulations, Experiences, and Prevention

**DOI:** 10.3390/s26031077

**Published:** 2026-02-06

**Authors:** Sasa Sladic, Even Zivic

**Affiliations:** 1 University of Rijeka, 51000 Rijeka, Croatia; 2 Faculty of Engineering, Juraj Dobrila University of Pula, 52100 Pula, Croatia; evenzivic@gmail.com

**Keywords:** PV inverters, smart inverters, electric power system, stability issues, simulations, blackout

## Abstract

**Highlights:**

Electric power systems with high penetration of renewables are highly exposed to stability issues. This problem was less important 20 or 30 years ago because electric power systems were dominated by synchronous generators. Since then, the percentage of renewables in the total generated power has been neglected. Systems were stable because of the properties of electric machines. Now, the properties of systems are highly influenced by the software of power inverters, especially photovoltaic sources (PV inverters). In this study, simulations and analysis are introduced to explain the lack of stability and series of events that could cause blackouts. These rare, high-impact events are sometimes called “black swans”.

**What are the main findings?**

- A possible explanation for recent massive blackouts is offered.
- Simulations were conducted in order to support the hypothesis.

**What are the implications of the main findings?**

- Events prior to massive blackouts in modern power systems could be detected.
- Different configurations of power systems were compared in order to decrease the vulnerability of systems with high penetration of renewables, mainly photovoltaic sources.
- An alternative to large electric power systems in the form of energy communities is put forth.

**Abstract:**

Several blackouts have recently occurred in Europe and elsewhere. Blackouts are mostly the consequence of a series of events rather than a single event. Their intensity and frequency could be related to the stronger penetration of renewables into electric power systems. Although many different renewable power units may be installed, they all have some basic properties: their power is not consistent, and power inverters are used to connect renewables to electric power systems. Photovoltaic systems are the most typical representative of this large group of power sources. These devices have become more sophisticated over the past few years, allowing for the precise control of large photovoltaic fields. In this situation, all power converters act as one. This means that they could be turned on and off during short intervals. Furthermore, their power factor could be independently adjusted. These functions are desirable for small systems; however, their implications for stability at a larger scale are usually not considered. In this study, the stability issues of a system under the high penetration of renewables and a unique control system are investigated. The most prominent case of this influence is a high-impact rare (HR) event, also known as a “black swan”, which could cause a massive blackout in an electric power system.

## 1. Introduction

Power converters have been studied for decades from different perspectives. However, their impact on electric power system stability has not been a frequent research topic [1,2]. Recent events in Southwestern Europe (Spain, Portugal, and parts of France) and Southeastern Europe (parts of Croatia, Bosnia and Herzegovina, Montenegro, and Albania) showed that developed power systems could experience massive blackouts. An Iberian blackout occurred on 28 April 2025, and one occurred in the Dinaric region in the previous summer (21 June 2024). It has been assumed that a high percentage of energy from renewables could have contributed to such events [3,4]. However, no obvious explanation for the Iberian event appears to have been offered [5,6,7]. This could increase insecurity with the further penetration of photovoltaic sources, especially because a similar event was recorded ten months earlier [8]. It could be concluded that electric power systems may have previously unidentified problems due to some aspects of renewables having been neglected. This study aimed to contribute to filling that gap. There is a demand for an obvious explanation and means to effectively avoid critical stability issues, which will necessitate studying different aspects of this problem [9,10]. Furthermore, new research should be conducted on power converters’ properties [1,2,11,12] because older studies could not explain all recent events and offer preventative measures for the new generation of sensors [13,14,15,16]. Network organization [17,18,19,20,21,22,23], especially including energy communities [22,23], could offer a robust solution, preventing the negative impact of power converters on power system stability [24,25,26,27,28,29,30] and blackout issues [31,32,33,34,35,36]. Nor should cyberthreats [6], power transients [37], renewable penetration [38], electromagnetic compatibility issues [39], or especially atmospheric issues be excluded [40,41,42] because both recent events occurred during the warmer part of year—the seasonal temperature increase. In such a frame, simulations were conducted to offer a new interpretation of power converters’ properties [43,44,45,46,47] in the series of events known as the “black swan”.

## 2. Smart Inverter-Related Hypothesis on Recent Blackouts

According to several sources, a rapid decrease in power occurred prior to the Iberian blackout. This (unexpected) lack of power resulted in high demands on power transfer from distant areas or even neighboring countries. At a given moment, it was no longer possible for these demands to be met. The consequence was the islanding of specific areas where a blackout occurred. As previously stated, a blackout can occur for several reasons; however, this study investigated a hypothesis where smart power inverters, more precisely solar power inverters, represent the starting point for the later domino effect. The basic criteria for stability in an electric power system are equality of the generated (*P*_GEN_) and load power (*P*_D_):

(1) $$P_{GEN}(t)=P_{D}(t)$$

This condition has to be met for a constant frequency to be maintained in the AC network. However, in order to predict future blackouts or to model the event chains of past events, both more detailed criteria and more complex models need to be used, such as those involving the synchronous generator swing equation [48,49]. Under an imbalance according to Equation (1), the angle between the rotor and stator magnetic field could change or even the frequency in the electric power system could change. For this study, the conclusions presented in [49] are of special interest because the authors derived a relation between the chaotic behavior of electric power system variables during overload. In this paper, (technical) overload is not mentioned; however, temporary power impulses are frequent (periodical or more likely occasional in real life). This means that a different type of disturbance is present. It is apparent that neglecting the impact of demand on power equality (1) could hypothetically be the primary cause for a blackout, which could appear several minutes or even several seconds after the imbalance is occurred. Smart inverters could be synchronized to the electric power system’s frequency using several methods. However, they do not bring any additional stability to the power system because the electric generators have inertia related to their rotor mass. Emulating this property still seems to be challenging for power inverters in spite of their advanced algorithms. Furthermore, advanced algorithms enable more flexibility in electric power systems by enabling power factor correction or the generation of additional reactive power to improve the conditions for power transfer and distribution, which could influence stability. The suggested reasons for stability issues in electric power systems include the following:

1. Power converter algorithms with specific conditions in the electric power system;
2. Electromagnetic compatibility (EMC) issues;
3. Atmospheric phenomena;
4. Cyber or electromagnetic attacks.

Over the past decade, various reasons for blackouts have been highlighted [8]; however, the Iberian (and Dinaric) blackout seems to have involved different phenomenology [1,11]. The penetration of renewables has never been higher than now, so the influence of the power inverter on stability has to be strongly limited by including an HVDC intersection [10] or by the physical segmentation of the electric power system to energy communities [21,22]. Despite several ideas for future development, the current unified European system is vulnerable and new blackouts can be expected. Southern Europe seems to be more exposed; however, the same effects could be expected in the northern areas (e.g., Central Europe) because, historically and technologically, the electric power system is the same. In order to explore the neglected issue of smart power converters, simulations were conducted. However, it can be expected that, in reality, the effects would be even stronger than the simulated effects because no simulation model can precisely model a lack of symmetry (e.g., voltage distribution across approximately the same capacitors) or EMC issues in an electric power network. Furthermore, electric network models can rarely incorporate specific geographical properties (e.g., a river crossing), which could influence power lines (e.g., resulting in an additional voltage drop) and even introduce time delays (related to voltage drops and exponential transients) in control signals for displaced photovoltaic fields (or particular inverters). This means that, in reality, dispersed results (voltage drop, time delay, EMI) could be expected compared to the quite-uniform simulation results.

These more distributed results indicate a variety of conditions that could promote a high-impact rare event (HR). For instance, a change in demand for the generation of reactive power could coincide with electromagnetic issues (maybe even atmospheric), having different effects on the same PV field on different sides of the river. Analogous effects occur on a single PV panel due to the shading effect. However, larger PV fields are exposed to more complex phenomenology, especially when smart inverters are involved, which has become more frequent in recent years. In this study, power inverters (both single-phase and three-phase) were simulated with repeated changes in demand for the generation of reactive power. In this study, the power flow and possible power imbalance during different demands for reactive power were investigated. Such conditions could appear when induction motors are involved in an electric power network (a common situation) and their demand for reactive power (or even their number) is not the same.

**Figure 1:**
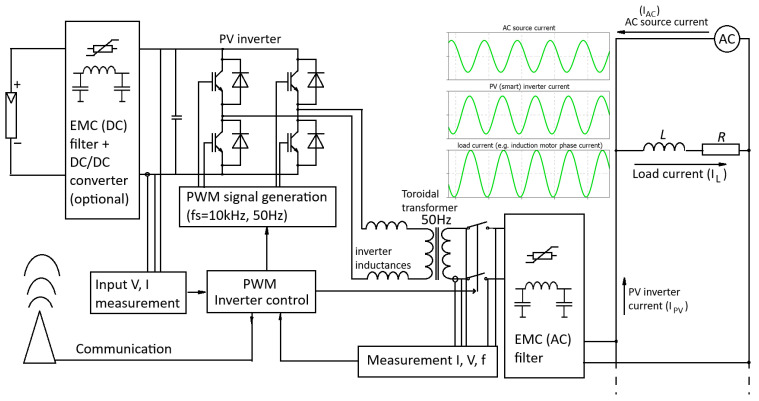
Power converter with reactive power control and three phase-shifted currents (AC generator, PV source, and load) in frame of IoT and (2).

Power converters can operate with an inductive or capacitive power factor (*pf*), which means that power flow in one case could be opposite to that in another case. The distortion of voltage and current signals could be less than 3% (THD < 3%) [43]. This means that photovoltaic sources could be emulated with pure sine waveforms [44,45,46,47] for both output voltage and supplied current (Figure 1). The addition of the AC source (motor-generator) current and PV source current (PV inverter) produces the load current:

(2) $$i_{AC}+i_{PV}+i_{L}=0$$

A power (PV) inverter can generate different waveforms of current (*i*_PV_) in order to compensate for different waveforms of load currents (*i*_L_). This means that impulse or even square wave currents could appear with rectifier addition. More sophisticated (e.g., bidirectional) power converters could be developed, to also maintain better power conditioning. Fluctuations of power appear in each of these cases. These fluctuations depend on previous energy accumulated in the system producing the potential imbalance related to (1). Smart inverters were not introduced to power systems in previous decades. Active power filters were designed to operate with a unity power factor, which meant that the reactive power flow during compensation was limited. Furthermore, the active power could change at the same moment, making the temporary power fluctuations more rapid. The time delays that can appear during the control of various photovoltaic fields do not improve the situation. Power converters were designed to achieve high performance in terms of reacting instantly to current conditions in the network and to control and protection signals. A new hypothesis could be synthesized—that the overlapping of different modes of operation introduced by smart power converters in combination with previous states of accumulated reactive power in the system could cause an effect similar to the shading of PV panels. This effect does not need to last long; a few seconds (a few power impulses) could be enough to induce a power imbalance in the system (1). This effect was spotted [12] during the demonstration of smart inverter properties, and as previously stated, it could hardly be noticed with the earlier generation of power inverters because they were operated with a unity power factor. Higher harmonics, which appear because of electrolysis or during the charging of electric vehicles, have a greater effect because of the reactive power related to higher harmonics.

## 3. Temporary Power Impulses as a Reason for Power Imbalance

In order to prove this hypothesis, it is important to analyze the smart converter’s operation during its different operation modes. A single-phase AC system was simulated because PV inverters are usually connected to such systems. Because the power in such a system is not constant, the temporary average power was observed. Therefore, a filter was included to make the changes in power in the frame of the AC system more visible:

(3) $$P=\frac{1}{T}\int_{t}^{t+T}p\left(t\right)\;dt$$

**Figure 2:**
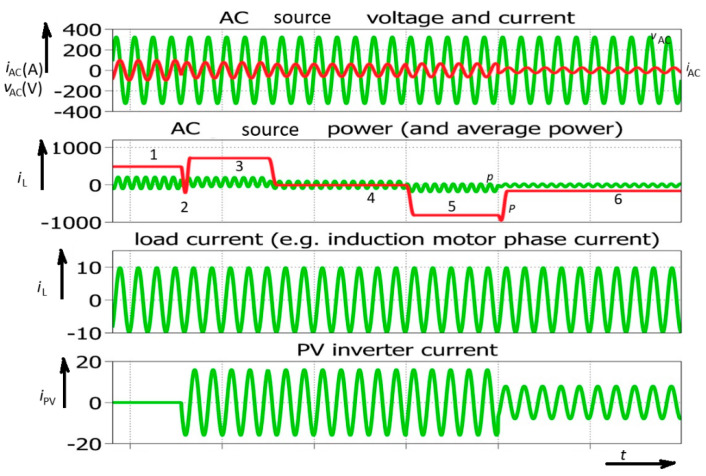
Power converter with reactive power control: simulated waveforms; *p*—temporary power of AC source (motor-generator); *P*—temporary average power of AC source (motor-generator). 100 ms/div.

Smart inverters are power inverters that incorporate properties of several earlier converter types, increasing their flexibility, but they introduce new stability issues in electric power systems. Figure 2 shows different modes of operation for a smart (bidirectional) inverter.

Four diagrams (Figure 2) of a smart inverter (including the AC generator/AC source, PV inverter, and load) provide basic information on the power flow in a simplified electric power system. In this case, a microinverter was studied. Its power typically does not exceed 1000 W (500 W was chosen for simulations). However, this type of PV inverter could be used as a building block for large PV fields. In order to analyze the phenomenology of an electric power system incorporating different power sources, including a large quantity of photovoltaic sources (microinverters), one selected PV inverter was analyzed, and the analysis was then expanded to the whole electric power system. At the beginning of the simulation, the PV (or more generally smart) inverter is not connected (its current equals zero). The AC generator (electric network) is supplying inductive load (e.g., a single-phase induction machine). During this interval, the AC generator (AC source) supplies 100% of the load power. This interval is marked with No. 1. (Figure 2). After the smart inverter is turned on, the AC generator power is increased to 700 W. This means that the smart inverter is acting as a rectifier consuming 200 W of power. At this point, it should be stated that PV inverters are not designed for such operation, but the analysis is not limited strictly to PV inverters; other renewables could also appear, e.g., electric vehicle chargers (or other bidirectional power converters). An obvious short interval of negative power also appears. This impulse-shaped power fluctuation has been marked with No. 2. In this interval, the AC source acts as a load because its power is negative. Because generators are generally not power loads, this electrical equipment was modeled as an AC source (also including electric storage) or it could simply be considered as an electric machine in a reversable power plant (motor-generator). Consequently, a synchronous generator could experience temporary speeding up (just to adjust the angle of its rotor magnetic field). This means that, despite the smart inverter acting as a load, a short interval appears where it acts as a generator. It could be concluded that the smart inverter acts in an uncontrolled manner despite its current being limited and the demanded phase-shift. This interval is followed by the next interval, where the smart (PV) inverter supplies the induction motor due to a phase change in its current. This means that the load power is compensated with the smart (PV) inverter, and the AC source power equals zero; *P*_AC_ = 0. This interval is marked with No. 4. Finally, the phase angle is further changed in order to demonstrate that the smart inverter is sending energy to the AC source. This interval can be recognized with negative AC source (generator) temporary power. As previously stated, it is presumed that the AC source represents a system that includes energy storage. Otherwise, the system will not be able to accumulate energy due to protection in a classic AC generator circuit [49,50]. It could be noted that transients of temporary power do not always appear. In order to achieve a temporary change in average power, it is necessary for the current’s direction to remain the same during the two consequent sine-wave half cycles. This is noted for the event marked with No. 2. It is important to notice the representative AC source current (*i*_AC_) waveform in that situation (Figure 2).

**Figure 3:**
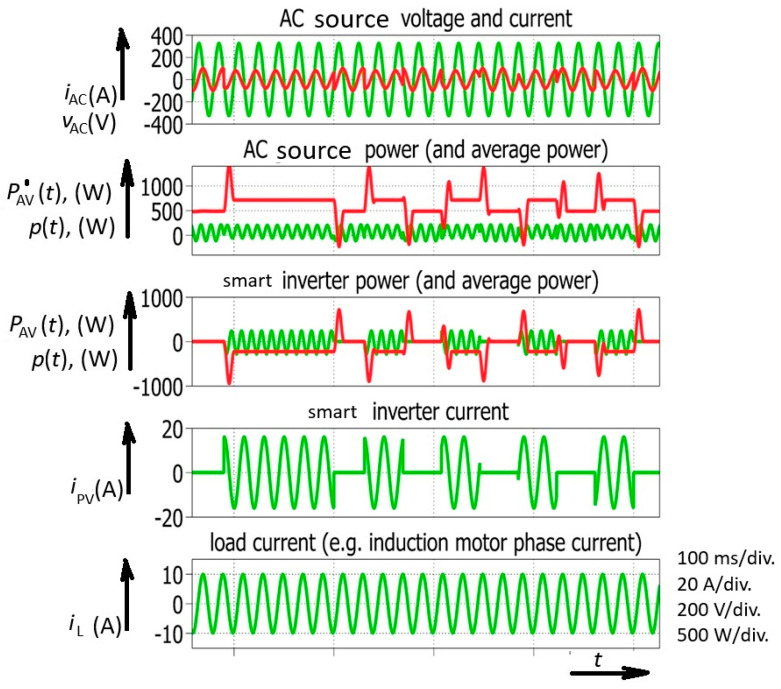
Simulated waveforms of smart power inverter with reactive power control including rapid solar power change (or successive turning on and off of the inverter).

After the analysis of the smart inverter’s flexibility, introducing the four-quadrant operation, a further step was taken in the study of the temporary average power changes. The temporary average power changes were exaugurated (frequent switching) in order to achieve a more detailed analysis (Figure 3). Upon analyzing the smart inverter current, sequential turning-on and turning-off could be observed.

**Figure 4:**
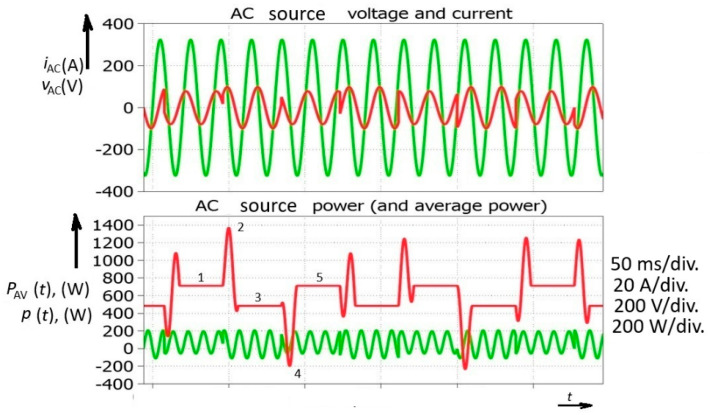
Power converter with reactive power control simulated waveforms including successive smart inverter phase-shift change.

Despite the fact that the load current is constant, the system experiences rapid changes in temporary average power, which is normally constant in the AC system during steady-state operation (Figure 3). These variations could be a consequence of a change in solar radiation. However, a change in solar radiation (or cloud appearance) is not the only reason for rapid temporary average power changes and stability issues in an electric power system with high penetration of smart (or PV) inverters. Reactive power generation brings even higher risk (Figure 4).

The demands on smart inverters include high performance and fast adaptation from one power level to another. In spite of the limited change in current amplitude (Figure 4), a power impulse (change in temporary average power) larger than 100% of generated power could appear. A simple calculation involving the average mean power in an AC power system is as follows:

(4) P=I·V·cos φ

where φ is the phase shift between the voltage (*V*) and current (*I*) phasors represented by their effective values, giving the theoretical limit of these power impulses. This means that the temporary power peak could theoretically reach a value of 200% of apparent power in a system only involving angle change. A change in the intensity of the current from the PV inverter could make this situation even more problematic (Figure 3). However, after numerous simulations, it seemed that the temporary average power impulse could be well estimated as 100% of the generated power.

These rapid changes involving a phase-shift change introduced by the smart inverter (the time base is 10ms in a 50 Hz AC system) could be observed in the next simulation (Figure 4). After the steady state, with the AC generator power reaching 700 W (No. 1), a power decrease was demanded. After a transient of power waveform, a new steady state was obtained at 500 W (No. 3). However, a peak power of 1400 W was reached before the transition to the new steady state was completed (No. 2). Another example could be observed when the AC source (generator) power had to be increased again to 700 W (No. 5). Before achieving the new steady state, the AC source experienced negative power (the motor-generator was receiving power), which meant that a synchronous generator somewhere in the electric power system was temporarily speeding up. The simulated value of the negative temporary average power was −200 W (No. 4). It could be concluded that a decrease in the power of the smart inverter brings about an increase in power for the AC source; however, in this particular case, an interval of negative power appears.

What does this mean for the power system? It means that “pressure” on the AC source will be transferred through the network. Another smart power inverter cannot compensate reactive power because it is under pressure from its own dynamics. Obviously, perturbation will travel through the electric network and the system could experience a dramatic decrease in power. A time delay that could be introduced in communication channels or could be the consequence of different threshold levels means that one power inverter could experience an increase in power and a neighboring PV inverter (if it reacts one millisecond later) could experience the same. These conditions are real because it is hard to imagine that large PV inverter fields (or charging stations) have completely the same parameters and achieve the same signals in the same fragment of the AC network period.

**Figure 5:**
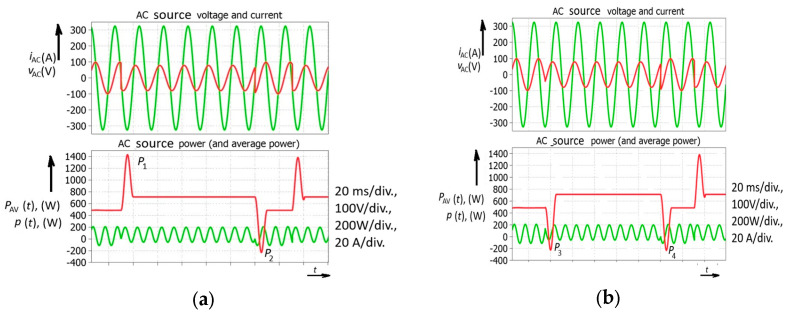
Simulated waveforms of voltages and currents for AC source and smart inverter with their temporary and temporary average powers: (**a**) positive power surge—*P*_1_; (**b**) negative power surge obtained by 5 ms delayed turning on of smart inverter—*P*_3_.

The rapid power drop and finally reversal of the power flow of the AC power source means that the electric power system is under high pressure due to this type of smart inverter (group) phenomenology. Figure 5 shows several possibilities that could occur in an electric power system, introducing the positive and negative spikes of normally constant single-phase temporary average power.

It should be noted that a unity power factor limits occurrences of this type. However, power factor changes may be necessitated by the different demands which occur in a network; for instance, demand for the generation of additional capacitive reactive power and an instant change in reactive power could occur.

**Figure 6:**
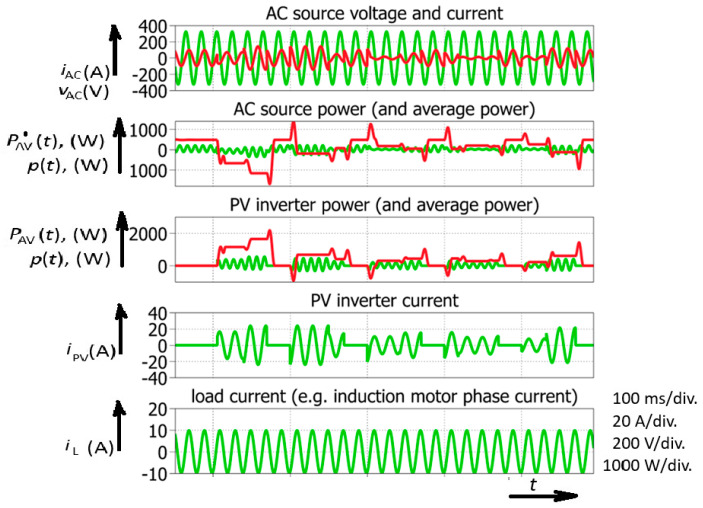
Simulated waveforms of PV power converter with reactive power control including rapid solar radiation change (or successive turning on and off of PV inverter).

Furthermore, considering the possibility that one PV field could generate positive energy and under the assumption that the control signal reaches the other field 5 milliseconds later, another field could temporarily act as a load (Figure 5). This means that AC currents flow between two PV fields, which could put the electric power system under additional pressure. It should be stressed that there are no transients (considering voltage and currents) in the system. The waveforms are ideal; only the power factor changes instantly. Power surges occurred for both the rectifier and inverter operation (Figure 6).

It could be observed that both positive and negative surges appeared again. The addition of an amplitude change for the PV inverter resulted in additional power surges (Figure 6). As stated before, the power surges could typically reach 100% of the generated power. That means demand on power could increase instantly.

The result of the interaction between a large number of power inverters could be a rapid loss of power over wide areas of the electric power system. The intention of this study was not to model the Iberian blackout; however, the intensive loss of power (15 GW in 5 s) reported by the media could be explained by the temporary average power flow simulated in this study. By coincidence, the percentage of PV-generated energy in Spain’s electric power system is 17% or 21.4 GW of installed power (44,520 GWh in 2024). The data provided for 2025 [4] includes 2/3 of total power (126 GW) generated by renewables (sun and wind). This means that power surges, which reach 100% of active (installed) power during sunny days, supplied by single-phase (or three-phase) inverters could bring about a temporary average power decrease of 20 GW at the national level in Spain. Theoretically, the synchronized action of all PV sources in each country could cause power surges in each country with installed PV fields: China, 1000 GW; USA, 200 GW; Germany, 117 GW; Italy, 36 GW; France, 24.85 GW; Greece, 10 GW; Austria, 9.4 GW; Switzerland, 8.17 GW; Hungary, 7.5 GW; Slovenia, 1.4 GW; Croatia, 0.87 GW. It is hard to expect that all power inverters would act as one; however, specific atmospheric conditions (traveling clouds, variations in solar power, or synchronized control signals, especially in the morning or evening) could result in such events. After an initial disturbance, the electric power system could show different responses including chaotic behavior [49].

It could be concluded that (single-phase) power converters could create high demand for power and act as a closed system for power exchange, producing bottlenecks and instabilities during the short intervals (few milliseconds).

**Figure 7:**
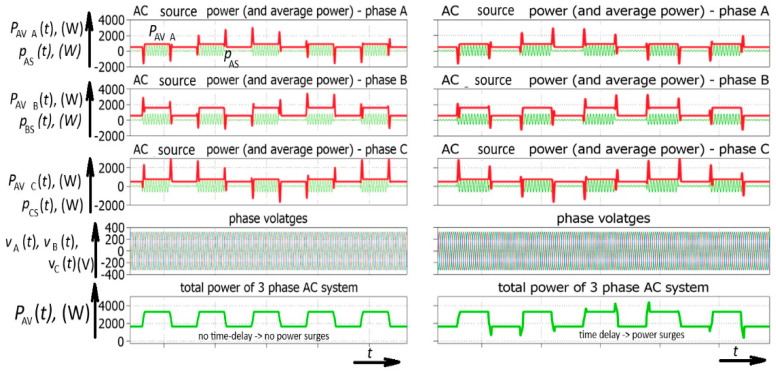
Simulated waveforms of temporary average power in three-phase system with three-phase power inverter, with no time delay and with time delay, in particular phase-control circuits (YY).

In the case of a three-phase inverter, a temporary power change appears in each phase. However, the total effect of these particular power bursts is that they compensate each other. This means that the transition of power between two states is linear (Figure 7). A three-phase system ideally filters these kinds of power transients introduced by its particular phases.

**Figure 8:**
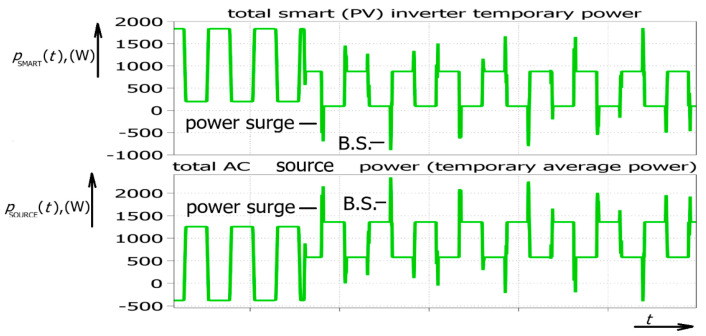
Simulated waveforms of total temporary AC source power *p*_SOURCE_(*t*) and total smart (PV) inverter temporary average power *p*_SMART_(*t*) recorded for three-phase inverter and 1ms and 2ms time delay for second and third inverter phase (500 W/div., 500 ms/div.).

However, if the time delay appears between different phases, the situation is different. This time delay could be the consequence of different threshold voltages introduced by different transistor drivers or variations in resistance in different control lines. It is important to mention that there is no phase shift between the ideal and delayed signal. The only difference between them is that the delayed signal is connected to the power system later (between 0 and 2 milliseconds later). This means that the power transients of particular phases do not appear at the same moment. As stated before, only a few milliseconds is needed. After the introduction of a time delay in different phase signals, a power imbalance occurs again (Figure 8).

As a consequence, the total power of the AC generator obtains a higher maximum power. These temporary power extremes (power surges) appear both during power increases and power decreases, which could be the consequence of solar energy radiation or the application of some type of bidirectional power converter, including an electric vehicle charger, an active power filter-related device, or some other renewables. In other words, the possible reasons for such temporary power bursts include both a change in smart inverter current amplitude and a change in the phase shift of the inverter current. This means that the primary source of power fluctuations, by definition, is single-phase smart inverters. Three-phase systems are made up of single-phase systems, which means that the same waveforms appear in three-phase systems; however, due to the properties of a three-phase system, these power bursts could be compensated between different phases. Furthermore, nonidealities could appear in three-phase inverters in the form of different threshold voltages and delays in their communication channels, which means that it cannot be excluded that temporary power bursts could also appear in three-phase inverters. Nevertheless, single-phase inverters are more important for sustaining the hypothesis of temporary power bursts as one of the reasons in the sequence of events causing blackouts, similar to the Iberian blackout, characterized by power loss that could hardly be compensated by the additional national electric power system of a country the size of Austria or Hungary. The most powerful power surge obtained by the time delay between particular phases of an inverter is marked with B.S., which does not necessarily mean that the electric power system is going to experience a “black swan”, but it is presumed that the combined action of single-phase and three-phase inverters contributed in such an event in Spain and surrounding countries.

## 4. Commercial Power Inverters and Their Reliability Issues

Commercial power converters (Table 1) are a rare subject of scientific papers; however, for this hypothesis, they are more important than any power inverter prototype. These converters can be found in photovoltaic systems all over the world in large numbers, and their change in performance due to temperature or other reasons could support the suggested hypothesis. These power inverters are commercial products generally made with cost-effective components. In addition, it is known that (electrolytic) capacitors usually limit the lifespan of this type of power electronic equipment. Furthermore, these capacitors are sensitive to temperature changes, especially those larger than 10 °C [51]. At this point, it is pertinent to remember that capacitors can be found in the majority of industrial transistor drivers, which could support the phenomenology shown in Figure 8 [52,53].

## 5. Network Configurations, Sensors, and Prevention

An instant phase shift appears at the output of smart converters used in photovoltaics and particularly for electric vehicle charging without the appearance of any overcurrents or overvoltages. This is probably why recent blackouts seem to be “mysterious” in their origin. Atmospheric phenomena could be the reason for the synchronization of power converter action. The reason for recent blackouts is purely mathematical, involving smart (or PV) inverters that react fast with ideal waveforms (THD < 3%). It is not easy to sense a sudden change in the phase shift of a smart inverter’s generated current. However, this is not the first time that demand for the sensing of a fast change in current has appeared. For instance, semiconductor components called thyristors, which have been almost completely replaced by modern (MOSFET or IGBT) transistors, have small thermal capacity. They can be destroyed by fast current changes. An optimal solution would be a microprocessor-based solution with the possibility of the fast calculation of current change, including local fast electronic switches. However, this approach is too slow for thyristor protection. The only way to protect thyristors is using fast fuses. After the fuse’s activation, a new fuse has to be inserted. It is obvious that such an approach is not very convenient for applications in electric power systems with frequent disruptions. The development of sensors for such applications will be a quite-interesting task and target for future investigation.

**Figure 9:**
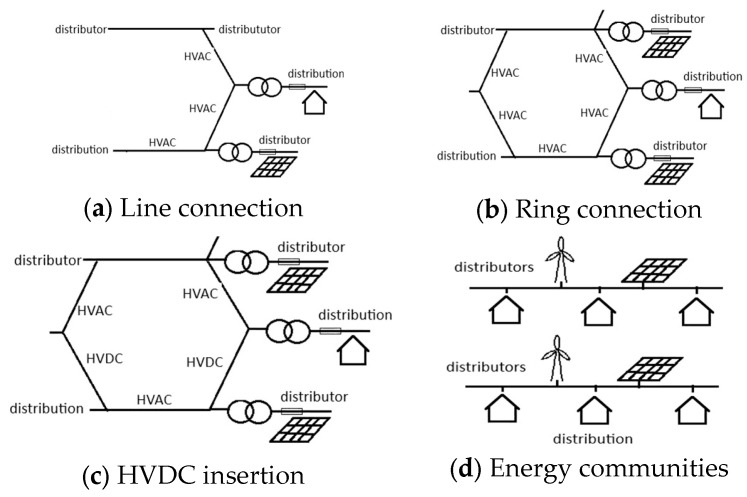
Electric power system configurations.

The limited possibilities for protection in electric power systems could be the reason for prevention in the form of different network configurations (Figure 9). The proper selection of an electric power system configuration can limit the negative effects of the deep penetration of renewables, especially when smart inverters are used. At present, it seems that prevention remains the most powerful tool for limiting the influence of the inconsistency of renewables. Several network configurations were analyzed:

The first case (Figure 9a) is a linear electric network. This configuration is very frequent in many countries, but it can hardly sustain the higher penetration of renewables. In the next case (Figure 9b), higher supply security was achieved considering the standard view of reliability (a system dominated by thermo-power plants and nuclear power plants). A new approach to reliability, which means reliability with high penetration of renewables including smart inverters, does not offer any additional reliability compared to the first case (Figure 9a). This is not applicable to an electric network with the insertion of direct current segments including the HVDC interconnection. Direct current systems, because of their DC links, could sustain temporary loss of power that could be a problem in an AC network; however, connection with AC systems is problematic for DC networks. It seems that the only sustainable solution involves small independent systems known as energy communities. Only in this case could large blackouts such as the Iberian blackout be avoided (Figure 9d). Furthermore, energy communities could be based on renewables following the decarbonization trend in the energy sector [22,23,54]. The availability of batteries and DC link capacitors could make these systems stable. It could be concluded that the 20th century belonged to AC currents; however, after recent events and conclusions, it is more likely that the 21st century will embrace DC current solutions more. Global AC electric power systems were made, interconnecting complete continents. However, in the future, local, probably DC, systems will be more important because of the lower probability of high-impact rare (HR) events.

## 6. Discussion

During the investigation of high-impact rare (HR) events related to photovoltaic (PV) systems, a courage hypothesis was taken into account. It has been assumed that a power system could be disturbed by a sudden change in (temporary average) power. Simulations were performed in order to prove that a sudden change in phase between the generated voltage and current could generate such HR events. After this, the hypothesis was investigated through numerous simulations and the literature was analyzed in order to find possible reasons for this occurrence. No obvious reason was found in the literature for the sudden decrease in power that could bring about a blackout in the following seconds. The conducted simulations elucidate various situations, which should be scaled to larger power. Time delays in smart inverter control seem to be an important element for sudden blackout occurrence.

At the beginning of the 21st century, a large part of inverter research targeted power inverters with a unity power factor (*pf*). Later, after large power photovoltaic applications, new functionalities were added to PV inverters [44,45,46,47]. Power inverters achieve additional flexibility, including the generation of inductive and capacitive reactive power. After that, blackouts appeared in places where they were not expected, without an obvious explanation. Furthermore, these events could not be related to similar events several years earlier.

Both mentioned blackouts (Iberian and Dinaric recent events) appeared in the morning during the sunny days when the heating up of power lines (but also the heating of the control circuits of microinverters) was intensive. According to the presented hypothesis, uniform heating up is not related to power oscillation. However, unequal heating up strongly supports this hypothesis because of the selective change in resistances in the frame of communication channels and driver circuits for particular smart inverters. This means that a nonuniform additional time delay could (and probably did) happen, increasing the time difference between the action of particular smart (or PV) inverters. According to the introduced hypothesis and conducted simulations, there is a “breaking point” where particular PV inverters could, respectively, start to generate power that cancels the produced energy.

Related to photovoltaic system shading, this effect could be called electronic system shading. In such a situation, one PV inverter could act against another PV inverter, or one PV field could even “compensate” the energy produced from another PV field. According to accessible data, an instantaneous decrease in produced power was exactly what happened prior to the recent blackouts.

Is it possible to develop such smart inverters that could act as synchronous generators? PV inverters are electronic and digital systems that could emulate analogous synchronous generators to some extent. However, in an electronic system, some bit in the system could change to make a “singularity”. Such an occurrence is unknown in an analogous device: the “old fashioned” synchronous generator. According to this hypothesis, DC systems are resilient to “electronic shading” but they could be influenced by events in larger AC power networks.

## 7. Conclusions

A possible explanation for the phenomenology of recent blackouts has been offered. Both single-phase and three-phase power inverters could introduce short-term power surges in electric power system. These power surges could reach 100% of installed power, and a large number of PV power converters could experience the same conditions or control signals at the same moment or with a short time delay (typically 1 millisecond). This means that a large number of PV power converters could act together (e.g., in the morning), introducing power surges into the electric power system. According to the hypothesis, distant power inverters could generate power of different signs, resulting in a rapid decrease in system power in the frame of the same photovoltaic field and further including other sources connected to the electric power system via smart inverters. To confirm this hypothesis, several simulations were performed. The initiation of such an event could be a signal for a change in reactive power. If this signal is delayed and does not reach all targeted PV inverters at the same time, all variations in cases are possible. The consequence could be that one converter is producing power and another smart inverter is consuming the same power. This effect could be named “electronic shading” in analogy with the photovoltaic shading known from solar cell theory. Despite the supporting simulations, the proposed hypothesis was not completely proven; however, it could provide a new perspective on some recent events. It is expected that this paper will support similar research, which could result in more reliable electric power systems.

This means that large electric power systems should be protected by additional sensors, enabling the possibility of disconnection from other parts of the system. The insertion of HVDC systems in AC transmission systems is one means of protection. Other possibilities for ensuring a stable electric supply (with a high percentage of renewables) involve a large number of small independent systems including a variety of electric power sources, known as energy communities.

## Figures and Tables

**Table 1:** Examples of commercial single-phase and three-phase inverters, sometimes with internal energy storage for sustaining bidirectional power flow (e.g., Figure 2).

Company	Title 2	Power (W), Energy (kW/h), Efficiency (%)	Phases
Maruson, Brea, CA, USA	Off-grid inverter SS3-HV1524M Off-grid inverter SS3-HV3024M	90–93% 1.5 kW 90–93% 3 kW	Single-phase Single-phase
Chasun Solar, Shang Rao China	Microinverter NEO 2000M-X Solar On-grid Solar Power HYX Microinverter On-grid	2 kW 1.6 kW, 1.8 kW, 2 kW 99.8% efficiency	Single-phase Single-phase
Qianneng International Trade, Wuxi, China	SUN-MI130/160/180/200/220 G4-EU	1.3–2 kW, 96.5% efficiency	Single-phase
Marstek, Xiangxi, China	Microinverter, Marstek MI800, WLAN, On-grid 2.5 kW Bidirectional On-Grid Power *	800 W Peak power 3 kW, 2.56 kWh, 15.36 kWh *	Single-phase Three-phase

* Power inverter, which enable bidirectional power flow, have commercial description “energy storage”, which means that both the inverter and batteries are included.

## Data Availability

The original contributions presented in this study are included in the article. Further inquiries can be directed to the corresponding author.

## Abbreviations

The following abbreviations are used in this manuscript:

EES	Electronic Energy System
HVAC	High-Voltage Alternate Current
HVDC	High-Voltage Direct Current
HR IoT	High-Impact Rare Event (“black swan”) Internet of Things
PF	Power Factor
PV	Photovoltaic
THD	Total Harmonic Distortion
MOSFET	Metal Oxide Semiconductor Field-Effect Transistor
IGBT	Insulate Gate Bipolar Transistor

## References

[1] Li M., Zhang X., Fu X., Geng H., Zhao W. (2024). Stability Studies on PV Grid-connected Inverters under Weak Grid: A Review. Chin. J. Electr. Eng..

[2] Zhang Q., Mao M., Ke G., Zhou L., Xie B. (2020). Stability problems of PV inverter in weak grid: A review. IET Power Electron..

[3] Freak Disappearance of Electricity Triggered Power Cut, Says Spain PM Sánchez. https://www.politico.eu/article/spain-portugal-power-cut-europe-electric-grid-pedro-sanchez/.

[4] Red Electrica. https://www.ree.es/en/press-office/press-release/news/press-release/2025/01/renewable-energies-generated-56-per-cent-spains-electricity-mix-2024.

[5] Alexander S. (2025). Europe’s Largest Blackout Exposes Grid Vulnerability, A Story About Renewables, Grid Inertia, Cross Boarder Capacities and More. https://www.thecommoditycompass.com/p/europes-largest-blackout-exposes.

[6] Lucas Laurensen: Rules, Not Renewables, Might Explain the Iberian Blackout. https://lucaslaursen.com/rules-not-renewables-might-explain-the-iberian-blackout/.

[7] Vialko D. Cyberattack May Be Behind Europe’s Largest Blackout in History: What We Know. RBC Ukraine.

[8] Albanija, Crna Gora, Bosna i Hrvatska Suočavaju se s Nestankom Struje. https://insajderi.org/hr/Albanija--Crna-Gora--Bosna-i-Hrvatska-suo%C4%8Davaju-se-s-nestankom-struje/.

[9] Sharma N., Acharya A., Jacob I., Yamujala S., Gupta V., Bhakar R. Major Blackouts of the Decade: Underlying Causes, Recommendations and Arising Challenges. Proceedings of the 2021 9th IEEE International Conference on Power Systems (ICPS).

[10] Makarov Y.V., Reshetov V.I., Stroev A., Voropai I. (2005). Blackout Prevention in the United States, Europe, and Russia. Proc. IEEE.

[11] Gomila D., Carreras B.A., Reynolds-Barredo J.-M., Martinez-Barbeito M., Colet P., Gomis-Bellmunt O. (2024). Reducing Blackout Risk by Segmenting European Power Grid with HVDC Lines. J. Mod. Power Syst. Clean Energy.

[12] Sladic S., De Santis M., Zivic E., Giernacki W. Smart PV Power Inverter for Emerging Solar Technologies: Case Study Perovskite Solar Cells. Proceedings of the MIPRO, IEEE Croatian Section.

[13] Lutzen H., Müller J., Kaminski N. A Review of Current Sensors in Power Electronics: Fundamentals, Measurement Techniques and Components to Measure the Fast Transients of Wide Bandgap Devices. Proceedings of the 2023 25th European Conference on Power Elec-tronics and Applications (EPE’23 ECCE Europe).

[14] Li P., Fan Y., Liu Z., Tian B., Wang Z., Li D., Han Z., Zhang Z., Xiong F. (2024). Application status and development trend of intelligent sensor technology in the electric power industry. IET Sci. Meas. Technol..

[15] Kunicki M., Fulneček J., Rozga P. (2024). Sensors and Fault Diagnostics in Power System. Sensors.

[16] Patin N. Power Electronics Applied to Industrial Systems and Transports, Sensors for Power Electronics.

[17] Liu K., Sheng W., Wang S., Ding H., Huang J. (2023). Stability of distribution network with large-scale PV penetration under off-grid operation. Energy Rep..

[18] Impram S., Nese S.V., Oral B. (2020). Challenges of renewable energy penetration on power system flexibility: A survey. Energy Strategy Rev..

[19] Desai J.P. Analysis of Power Swings and Blackouts. Proceedings of the 2021 IEEE Congreso Estudiantil de Electrónica y Electricidad (INGELECTRA).

[20] Gholami A., Shekari T., Amirioun M.H., Aminifar F., Amini M.H., Sargolzaei A. (2018). Toward a consensus on the definition and taxonomy of power system resilience. IEEE Access.

[21] Chen C., Wang J., Ton D. (2017). Modernizing distribution system restoration to achieve Grid resiliency against extreme weather events: An integrated solution. Proc. IEEE.

[22] Yiasoumas G., Psara K., Georghiou G.E. A review of Energy Communities: Definitions, Technologies, Data Management. Proceedings of the 2022 2nd International Conference on Energy Transition in the Mediterranean Area (SyNERGY MED).

[23] Nykyri M., Annala S., Honkapuro S., Silventoinen P. Review of Energy Communities in Agriculture. Proceedings of the 2024 20th International Conference on the European Energy Market (EEM).

[24] IEEE PES Industry Technical Support Leadership Committee Impact of IEEE 1547 Standard on Smart Inverters and the Applications in Power Systems. https://www.nrel.gov/media/docs/libraries/grid/smart-inverters-applications-in-power-systems.pdf.

[25] Rajiv K.V. (2022). Smart Inverter Functions. Smart Solar PV Inverters with Advanced Grid Support Functionalities.

[26] Xue Y., Starke M., Dong J., Olama M., Kuruganti T., Taft J., Shankar M. On a Future for Smart Inverters with Integrated System Functions. Proceedings of the 2018 9th IEEE International Symposium on Power Electronics for Distributed Generation Systems (PEDG).

[27] Priyadarshi N., Sanjeevikumar P., Azam F., Bharatiraja C., Singh R. (2023). Advanced Power Electronics, Converters for Future Renewable Energy Systems.

[28] Gonzalez-Longatt F.M., Torres J.L.R. (2021). Modelling and Simulation of Power Electronic Converter Dominated Power Systems in PowerFactory.

[29] Kishan D., Kannan R., Reddy B.D., Prabhakaran P. (2023). Power Electronics for Electric Vehicles and Energy Storage—Emerging Technologies and Developments.

[30] Lei S., Chen C., Zhou H., Hou Y. (2019). Routing and scheduling of mobile power sources for distribution system resilience enhancement. IEEE Trans. Smart Grid.

[31] Stankovski A., Gjorgiev B., Locher L., Sansavini G. (2023). Power blackouts in Europe: Analyses, key insights, and recommendations from empirical evidence. Joule.

[32] Xu Y., Chi Y., Yuan H. (2023). Recent Large-Scale Blackouts in the World. Stability-Constrained Optimization for Modern Power System Operation and Planning.

[33] Venkatanagaraju K., Rakesh M., Yenugu B.C., Sharma P.P., Andanapalli K., Biswal M. Major Power System Blackouts: A Survey. Proceedings of the 2024 10th International Conference on Electrical Energy Systems (ICEES).

[34] Zhao Q., Qi X., Hua M., Liu J., Tian H. Review of the recent blackouts and the enlightenment. Proceedings of the CIRED 2020 Berlin Workshop (CIRED 2020).

[35] Fita N.D., Utu I., Marcu M.D., Pasculescu D., Mila I.O., Popescu F.G., Lazar T., Schiopu A.M., Muresan-Grecu F., Cruceru E.A. (2025). Global Energy Crisis and the Risk of Blackout: Interdisciplinary Analysis and Perspectives on Energy Infrastructure and Security. Energies.

[36] Haes Alhelou H., Hamedani-Golshan M.E., Njenda T.C., Siano P. (2019). A Survey on Power System Blackout and Cascading Events: Research Motivations and Challenges. Energies.

[37] Li X., Li Z., Guan L., Zhu L., Liu F. Review on Transient Voltage Stability of Power System. Proceedings of the 2020 IEEE Sustainable Power and Energy Conference (iSPEC).

[38] Fernández L. (2025). Solar Photovoltaics in Europe—Statistics & Facts, Statista. https://www.statista.com/topics/5088/solar-photovoltaic-industry-in-europe/#topicOverview.

[39] Yang F., Dan Z., Pan K., Yan C., Ji X., Xu W. (2024). ReThink: Reveal the Threat of Electromagnetic Interference on Power Inverters. arXiv.

[40] Chojnacki A.Ł. (2025). Modelling the influence of atmospheric conditions represented by wind, precipitation and air temperature on the intensity of failure and restoration time of medium-voltage power lines. Sustain. Energy Grids Netw..

[41] Nicoll K., Harrison R., Barta V., Bor J., Brugge R., Chillingarian A., Chum J., Georgoulias A., Guha A., Kourtidis K. (2019). A global atmospheric electricity monitoring network for climate and geophysical research. J. Atmos. Sol. -Terr. Phys..

[42] Rycroft M.J. (2025). Some Recent Key Aspects of the DC Global Electric Circuit. Atmosphere.

[43] Zajec P., Voncina D. Overview and development of design concepts of AC power sources for measuring test equipment. Proceedings of the 2013 International Conference-Workshop Compatibility and Power Electronics.

[44] Javed A.H., Nguyen P.H., Morren J., Slootweg J.H. Using Smart PV Inverters for Reactive Power Management in Distribution Grids. Proceedings of the 2023 IEEE Belgrade PowerTech.

[45] Aboshady F.M., Pisica I., Zobaa A.F., Taylor G.A., Ceylan O., Ozdemir A. (2023). Reactive Power Control of PV Inverters in Active Distribution Grids With High PV Penetration. IEEE Access.

[46] Wang F., Tuluhong A., Luo B., Abudureyimu A. (2025). Control Methods and AI Application for Grid-Connected PV Inverter: A Review. Technologies.

[47] Lulbadda K.T., Hemapala K.U. Novel Concepts for Additional Functions of Smart PV Inverters. Proceedings of the 2020 IEEE International Conference on Computing, Power and Communication Technologies (GUCON).

[48] Waskito F., Wijaya F.D., Firmansyah E. (2025). Review of Virtual Inertia Based on Synchronous Generator Characteristic Emulation in Renewable Energy-Dominated Power Systems. Electricity.

[49] Premnath B., Sofroniou A. (2025). Analysing Load Shedding to Increase Stability in the Swing Equation. Mathematics.

[50] Al-Najjar H., Pfeifer C., Al Afif R., El-Khozondar H.J. (2022). Performance Evaluation of a Hybrid Grid-Connected Photovoltaic Biogas-Generator Power System. Energies.

[51] 71 Power Inverter Manufacturers in 2025. https://us.metoree.com/categories/inverter/.

[52] Balogh L. Bootstrap Circuitry Selection for Half Bridge Configurations (Rev. A) Texas Instruments. https://www.ti.com/lit/ml/slua618a/slua618a.pdf?ts=1767323364630.

[53] Yamaguchi D., Cheng Y.S., Mannen T., Obara H., Wada K., Sai T., Takamiya M., Sakurai T. (2022). An Optimization Method of a Digital Active Gate Driver Under Continuous Switching Operation Being Capable of Suppressing Surge Voltage and Power Loss in PWM Inverters. IEEE Trans. Ind. Appl..

[54] Viskovic A., Franki V., Jevtic D. Artificial intelligence as a facilitator of the energy transition. Proceedings of the 2022 45th Jubilee International Convention on Information, Communication and Electronic Technology (MIPRO).

